# Evaluation of High-Efficacy Insecticides and Control Efficacy Using UAV Application Against the Sea Buckthorn Fruit Fly, *Rhagoletis batava obscuriosa*

**DOI:** 10.3390/insects17040380

**Published:** 2026-04-01

**Authors:** Yang Zhou, Jipeng Jiao

**Affiliations:** 1 College of Forestry and Landscape Architecture, Xinjiang Agricultural University, Urumqi 830052, China; zy19834545375@163.com; 2 Key Laboratory of Integrated Pest Management on Crops in Northwestern Oasis, Ministry of Agriculture and Rural Affairs, Xinjiang Key Laboratory of Agricultural Biosafety, Institute of Plant Protection, Xinjiang Academy of Agricultural Sciences, Urumqi 830091, China

**Keywords:** *Rhagoletis batava obscuriosa*, Tephritidae, insecticide screening, unmanned aerial vehicle (UAV), field efficacy

## Abstract

Aiming at addressing the lack of efficient, low-toxicity insecticides and precise application technologies in the current control of *R. batava obscuriosa*, this study intends to screen high-efficacy insecticides for UAV application and evaluate their field control effects, so as to provide a reference for the field control of *R. batava obscuriosa*. The residual film method was used to determine the laboratory toxicity of seven insecticides to adult *R. batava obscuriosa*. The LC_50_ and LT_50_ values of the seven insecticides were calculated. Based on these results, four high-efficacy insecticides were selected for UAV field efficacy testing. The control effects of different insecticides were evaluated through indicators such as population reduction rate, corrected control effect and fruit infestation rate after application. Laboratory toxicity results showed that 10% abamectin·beta-cypermethrin exhibited the strongest toxicity at 24 h (LC_50_ = 22.108 mg/L). At 48 h after application, 5% emamectin benzoate was optimal (LC_50_ = 5.486 mg/L). At 72 h after application, 10% broflanilide (LC_50_ = 1.796 mg/L) and 10% abamectin·β-cypermethrin (LC_50_ = 2.665 mg/L) performed best. Field test results indicated that the population reduction rate of 10% abamectin·β-cypermethrin reached 91.73% at 7 days after application, while the other three insecticides had population reduction rates above 80%, showing good overall control effect; at 15 days after application, 20% chlorfluazuron-thiamethoxam had the highest corrected efficacy (80.91%), and the fruit infestation rate of each insecticide group (24.75–27.49%) was significantly lower than that of the control group (58.08%). All four selected insecticides can effectively control the population of *R. batava obscuriosa* adults and can be used as quick- and long-acting insecticides against *R. batava obscuriosa* in production. Combined with UAV application technology, they can achieve efficient control of *R. batava obscuriosa*.

## Simple Summary

As an economically vital tree species in China, sea buckthorn (*Hippophae rhamnoides*) has extensive planting areas and substantial industrial value. However, the expansion of *H. rhamnoides* cultivation has been accompanied by severe infestations by the pest *Rhagoletis batava obscuriosa*, causing substantial damage to sea buckthorn fruits and hindering the development of the sea buckthorn industry. This study aims to screen high-efficacy insecticides and test their control efficacy against *R. batava obscuriosa* via unmanned aerial vehicle (UAV) application. The results revealed that all four selected insecticides exhibited excellent control efficacy, with the fruit infestation rate in each treatment group being significantly lower than that in the control group. This research lays practical groundwork for the effective and precise control of *R. batava obscuriosa*.

## 1. Introduction

Sea buckthorn (*Hippophae rhamnoides*), commonly known as vinegar willow or sour thorn, is a deciduous shrub or small tree of the genus *Hippophae* in the family Elaeagnaceae. It has a wide geographical distribution, mainly ranging from 20° E to 123° E and 27° N to 69° N, spanning cold temperate, temperate, subtropical alpine regions of Europe and Asia, and parts of North America [1]. China has the largest sea buckthorn planting area globally. By 2020, there were more than 30 million hm^2^ of sea buckthorn forests in 19 provinces and autonomous regions in Northern China [2]. In addition, the fruits and seeds of sea buckthorn, as ingredients in health products [3,4,5], have extremely high edible and medicinal values. Sea buckthorn is rich in various trace elements required by the human body, as well as hundreds of bioactive substances, such as vitamins, serotonin, proanthocyanidins, triterpenes, polyphenols, and flavonoids [6,7]. It has anti-aging, anti-radiation, anti-tumor, hypoglycemic, and other functions and can enhance human immunity and promote body fat metabolism [8,9,10].

Xinjiang is rich in sea buckthorn resources which are widely distributed. There are natural sea buckthorn forests in 32 counties (districts) [11]. In recent years, with the expansion of sea buckthorn planting areas and changes in climate conditions, *Rhagoletis batava obscuriosa*, a pest that damages sea buckthorn fruits, has spread rapidly in major sea buckthorn-producing areas such as Xinjiang and Inner Mongolia, becoming the main pest endangering sea buckthorn fruits and causing serious losses to the sea buckthorn industry [12].

The genus *Rhagoletis* (Diptera: Tephritidae) comprises numerous fruit fly species of significant agricultural and economic importance worldwide. Members of this genus, such as the apple maggot (*Rhagoletis pomonella*), cherry fruit fly (*Rhagoletis cerasi*), and walnut husk fly (*Rhagoletis completa*), are notorious pests that cause extensive damage to commercial fruit crops by ovipositing into ripening fruits, rendering them unmarketable. Their larvae feed internally on fruit pulp, leading to premature fruit drop, reduced yields, and substantial economic losses [13,14,15,16]. Many *Rhagoletis* species are also subject to strict phytosanitary regulations, further amplifying their impact on global trade.

Currently, *R. batava obscuriosa* is mainly distributed in Nordic countries such as Russia, Germany, and Belarus [17]. It was first discovered in Jianping County, Liaoning Province, China, in 1985. In addition, it has occurred in many areas, such as Youyu in Shanxi, Yulin in Shaanxi, Heilongjiang, and Qinghai. It mainly damages sea buckthorn pulp by larvae, directly causing fruit damage and seriously affecting the commercial value of fruits [18].

The life cycle characteristics of *R. batava obscuriosa* poses significant challenges to its control. *R. batava obscuriosa* is univoltine, completing one generation per year, and overwinters as pupae in the soil beneath host plants. Adult emergence occurs from June to July, with the population peak occurring from mid-July to early August. Females exhibit an oviposition preference for fruits that have already begun to color. Typically, only a single oviposition scar is observed per infested fruit. In untreated orchards, the fruit infestation rate can exceed 85%, resulting in near-total economic loss of marketable yield. Effective monitoring is essential for timely intervention. Currently, surveillance of *R. batava obscuriosa* is conducted using yellow sticky traps, with research indicating that traps with a wavelength of 570 nm exhibit the highest trapping efficiency. In practice, traps (typically 20 cm × 25 cm) are positioned within the sea buckthorn canopy at a standard height of approximately 1.5 m and inspected at 3–7 day intervals during the adult flight period. This surveillance enables determination of first adult catch—which signals the optimal timing for insecticide application based on occurrence phenology—and facilitates tracking of population dynamics to inform control decisions. In addition to visual attraction, chemical attractants play a crucial role in fruit fly monitoring and control. Ammonium carbonate and ammonium acetate have demonstrated strong attractiveness to *R. batava obscuriosa* adults. Among synthetic attractants, lure TQ exhibits the highest trapping efficacy and longest field persistence [19,20,21,22].

Despite these advances in understanding the biological characteristics and geographical distribution of *R. batava obscuriosa*, significant deficiencies remain in the systematic screening of high-efficacy, low-toxicity insecticides and the development of precise application technologies. Unmanned aerial vehicles (UAVs) have emerged as a transformative technology in modern pest management, offering distinct advantages for precision application in complex terrains such as sea buckthorn forests, including rapid coverage, improved canopy penetration, reduced chemical usage, and lower labor requirements. Despite these proven benefits for other agricultural systems, the potential of UAV technology for controlling *R. batava obscuriosa* remains largely unexplored, highlighting the need for integrated approaches combining effective insecticides with precision delivery systems.

Therefore, conducting research on insecticide screening and field UAV precise application technology is of great theoretical and practical significance for improving the control effect of *R. batava obscuriosa*, reducing economic losses, and ensuring the sustainable development of the sea buckthorn industry.

With the aim to solve the above problems and meet the actual needs of prevention and control of the sea buckthorn industry, this study systematically determined the laboratory toxicity (LC_50_ and LT_50_) of seven insecticides to adults of *R. batava obscuriosa* and clarified the differences in the quick- and long-acting properties of different insecticides. Based on this, four high-efficacy insecticides were selected, and field efficacy verification tests were conducted using plant protection UAVs. A comprehensive evaluation was performed based on core indicators including population reduction rate, corrected control efficacy, and fruit infestation rate. This study provides a theoretical basis and technical support for the effective and precise control of *R. batava obscuriosa*, which holds great significance for promoting the high-quality development of the sea buckthorn industry.

## 2. Materials and Methods

### 2.1. Laboratory Toxicity Determination of Seven Insecticides Against R. batava obscuriosa

#### 2.1.1. Insect Source

**Figure 1:**
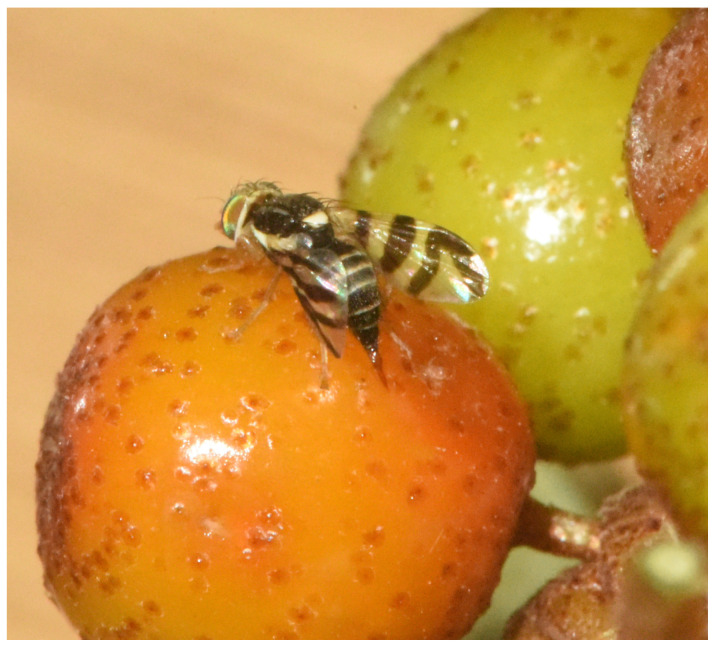
Adult morphology of *Rhagoletis batava obscuriosa* (photographed by Jipeng Jiao in Urumqi County).

In mid-June 2024, before the emergence of *R. batava obscuriosa*, overwintering pupae were collected from the soil of infested sea buckthorn groves in Qinghe County, Altay Prefecture, Xinjiang. The pupae were transported to the laboratory and placed in insect rearing cages (40 cm × 40 cm × 40 cm). Following emergence under ambient laboratory conditions, adults were identified as *R. batava obscuriosa* based on morphological characteristics (Figure 1): the pronotum is light yellow with three dark brown stripes medially, the scutellum is white, and the wings are transparent with four black to yellowish-brown transverse bands, the fourth of which is V-shaped. The adults were then transferred to disposable plastic containers with ventilation holes in the lids covered with gauze, and were reared and observed on a diet consisting of sucrose and yeast powder at a 3:1 ratio.

#### 2.1.2. Insecticides

In this experiment, seven insecticides were selected, including: 10% broflanilide (Zhejiang Shuoguo Shidai Agricultural Technology Co., Ltd., Hangzhou, China), 10% abamectin·beta-cypermethrin (Yantai Shenghai Agriculture Co., Ltd., Yantai, China), 20% chlorfluazuron-thiamethoxam (Guangzhou Yitian Biotechnology Co., Ltd., Guangzhou, China), 10% lambda-cyhalothrin (Juancheng County Hongfeng Agricultural Technology Co., Ltd., Heze, China), 6% spinetoram (Shandong Feixiang Agricultural Development Co., Ltd., Linyi, China), 5% emamectin benzoate (Hunan Nongda Haiten Agrichemical Co., Ltd., Yueyang, China), and 0.5% ivermectin (Taizhou Shunyi Co., Ltd., Taizhou, China).

#### 2.1.3. Test Methods

(1) Determination of LC50 of Seven Insecticides Against Adults of *R. batava obscuriosa*

The residual film method was employed. Based on the results of preliminary experiments, each test insecticide was diluted with an appropriate solvent (acetone or water) to prepare five concentration gradients (Table 1). The choice of solvent was determined by the formulation characteristics of each insecticide:

Specifically, 10% broflanilide (SC, suspension concentrate), 20% chlorfluazuron·thiamethoxam (SC), and 6% spinetoram (SC) were diluted with acetone. Suspension concentrates contain active ingredients that are insoluble or sparingly soluble in water, dispersed as fine solid particles in an aqueous medium. Acetone effectively dissolves these active ingredients, ensuring complete and uniform distribution in the test solution.

The remaining insecticides—10% abamectin·β-cypermethrin (EW, emulsion in water), 10% lambda-cyhalothrin (EW), 5% emamectin benzoate (ME, micro-emulsion), and 0.5% ivermectin (EC, emulsifiable concentrate)—are formulated with emulsifiers or solvents that allow them to readily dissolve or disperse in water. These were therefore diluted with water to better simulate field application conditions while maintaining test consistency.

Acetone and water were used as corresponding solvent controls to account for any potential effects of the solvents themselves on insect mortality. Preliminary experiments confirmed that there was no significant difference in mortality between the acetone control and the water control under the residual film method.

Each test insecticide was diluted with an appropriate solvent (acetone or water) to prepare five concentration gradients (Table 1), with each concentration replicated three times. After dilution to the desired concentrations, 5 mL of the diluted solution was transferred into a clean, dry residual film bottle. The bottle was rotated to ensure even coating of the inner wall with the solution, forming a residual film. Following complete volatilization of the acetone and water, the bottles were placed upright in a dry, well-ventilated area for 24 h to allow any residual organic solvent to fully evaporate, thereby preparing the residual film for subsequent use.

Adults of *R. batava obscuriosa* specimens exhibiting relatively consistent physiological status at five days post-emergence were selected as test insects. To facilitate transfer, the test insects were first subjected to mild anesthesia based on the methods described by Zhang [23] and Li [24] et al. Following preliminary experimental results, the adults were placed into a resealable plastic bag (30 cm × 20 cm). Subsequently, a cotton ball containing 2 mL of ether (applied via a dropper pipette) was quickly placed into the bag, which was then sealed. After 30–45 s of anesthesia (as determined by timing and observation of immobilization), the adults were allowed to fully recover at room temperature until regaining normal crawling ability (approximately 10 min). Subsequently, normally active adults were selected using a fine brush and transferred into the pre-prepared residual film bottles, with the bottle openings secured using prepared lids. Each bottle contained 10 adults (sex ratio 1:1), and each treatment was replicated three times. Control group insects, subjected to identical ether anesthesia and recovery procedures, were transferred into clean residual film bottles without insecticide treatment. The residual film bottles were placed horizontally in an illuminated incubator maintained at a temperature of 26 ± 1 °C, a relative humidity of 70% ± 5%, and a photoperiod of 14:10 (L:D) h for rearing. Behavioral responses of the adults following exposure to the insecticides were observed and recorded. Mortality of *R. batava obscuriosa* was assessed at 24, 48, and 72 h post-treatment. Individuals that failed to exhibit any movement upon gentle probing with a fine brush were considered dead, and the number of deceased adults was recorded at each time interval. Corrected mortality was then calculated for each treatment group using Abbott’s formula (Equation (1)).

(2) Determination of LT_50_ of Seven Insecticides Against Adults of *R. batava obscuriosa*

The residual film method was used to assess the effects of each tested insecticide on the longevity of *R. batava obscuriosa*. Based on the pre-test, the median lethal time for *R. batava obscuriosa* was determined according to the recommended concentration of each insecticide. The treatments were diluted 500, 1000, and 1500 times, with acetone and water as the control. Each treatments were repeated 3 times; ten adults (sex ratio 1:1) were introduced according to the method described in Section 1, marked on the residual film bottles, and placed in an artificial climate box for rearing. The number of dead test adults in each treatment was observed and recorded at 12, 24, 48, 72, and 96 h after treatment. For each treatment group, corrected mortality was calculated using Equation (1).

#### 2.1.4. Data Analysis

Mortality data were corrected using Abbott’s formula (Equation (1)). The corrected mortality data were then statistically analyzed using Excel 2019. The probit analysis module in SPSS 25.0 was employed to fit toxicity regression equations for the seven insecticides against R. batava obscuriosa. Based on this analysis, the median lethal concentration (LC_50_), median lethal time (LT_50_), 95% confidence intervals, and correlation coefficients were calculated for each insecticide. The goodness-of-fit of the probit model was assessed using the Pearson chi-square test, with *p* > 0.05 indicating an acceptable model fit.

Corrected mortality = (treatment mortality − control mortality)/(100 − control mortality) × 100%(1)

### 2.2. Field Control Test of Four Insecticides Against R. batava obscuriosa

#### 2.2.1. Insecticides

The following four insecticides were used in the field control trials: 10% broflanilide, 10% abamectin·β-cypermethrin, 20% chlorfluazuron-thiamethoxam, 10% lambda-cyhalothrin.

#### 2.2.2. Test Methods

Field control trials were conducted in areas with severe *R. batava obscuriosa* infestation in Qinghe County. The field trial employed plant protection UAVs for insecticide application, which was performed by professional technicians. The model of the plant protection UAV was DJI Agras T20 (DJI, Shenzhen, China), equipped with centrifugal nozzles, with an effective spray width of approximately 7 m, an operation height of 1.5 m–2 m above the sea buckthorn canopy, and a spray tank capacity of 30 L. The volume median diameter (VMD) of the spray droplets was controlled within the range of 150–250 μm. A water-only treatment was included as a spray control to account for any potential effects of the spraying procedure itself on insect populations. The four insecticides and the control were each replicated three times. Each experimental unit consisted of a 1600 m^2^ plot. The four insecticides were applied with water according to the recommended formulation dosage of 900 g/hm^2^, and a blank control was used.

Within each experimental unit, four yellow sticky traps were suspended diagonally at a height of 1.5 m, with a spacing of 10 m between adjacent traps. Based on continuous monitoring of adult population dynamics, the peak emergence period of *R. batava obscuriosa* was determined, and insecticide applications were scheduled prior to this peak to achieve effective control. The number of *R. batava obscuriosa* adults captured by the traps was recorded three days before application to establish the pre-treatment population baseline, and again immediately prior to application. After application, trap catches were documented at 3, 5, 7, 9, 12, and 15 d after treatment. At 7 and 15 d after application, 25 sea buckthorn trees were selected at each test site using the five-point method, with one branch selected and numbered per tree. For each replicate, 100 sea buckthorn fruits were randomly collected, and the number of infested fruits (those exhibiting larval exit holes or other feeding damage) was recorded. Fruits were considered infested if they exhibited larval exit holes or other visible feeding damage. The number of infested fruits was recorded for each sample, and the fruit infestation rate was calculated accordingly. Based on these data, the fruit infestation rate was calculated, and the control efficacy of each treatment was determined.

#### 2.2.3. Data Analysis

Excel 2019 and SPSS 25.0 were used to calculate the population reduction rate, corrected control effect (i.e., corrected population reduction rate), number of infested fruits per 100 sea buckthorn fruits, and infestation rate of *R. batava obscuriosa* after application. Before the analysis, Levene’s test for homogeneity of variance was used to determine whether the original data conformed to a normal distribution. The trapping amount and infestation rate data were analyzed using one-way analysis of variance (ANOVA), and Duncan’s test was used to compare the significant differences between variables (*p* < 0.05).

The fruit infestation rate, insect population reduction rate, and corrected control efficacy were calculated according to Equation (2), Equation (3), and Equation (4), respectively.

Fruit infestation rate = (Number of infested fruits surveyed/Total number of fruits surveyed) × 100%(2)

Insect population reduction rate = (Pre-treatment population − After treatment population)/Pre-treatment population × 100%(3)

Corrected control efficacy = (After treatment population reduction rate − Control population reduction rate)/(1 − Control population reduction rate) × 100%(4)

## 3. Results

### 3.1. Laboratory Toxicity Determination of Seven Insecticides Against R. batava obscuriosa

#### 3.1.1. LC_50_ of Seven Insecticides Against Adults of *R. batava obscuriosa*

**Figure 2:**
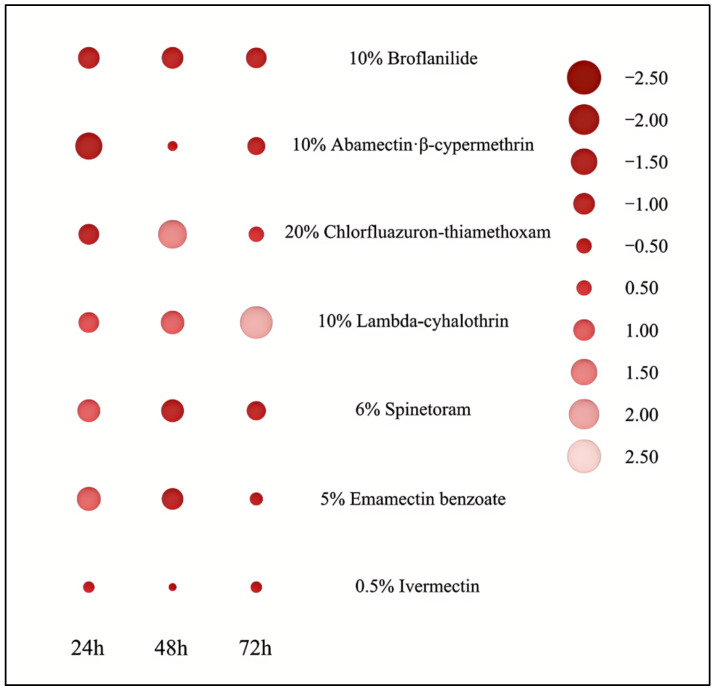
Comparison of the toxicity of seven insecticides following residual film treatment. (The darker the color block, the lower the LC_50_ value, indicating stronger toxicity).

As shown in Figure 2 and Table 2, 24 h after the application of the seven insecticides, 10% abamectin·β-cypermethrin had the strongest toxicity to *R. batava obscuriosa* adults, with an LC_50_ of 22.108 mg/L, followed by 10% broflanilide (25.712 mg/L) and 20% chlorfluazuron-thiamethoxam (26.237 mg/L). Among the seven tested insecticides, 5% emamectin benzoate exhibited the weakest toxicity to *R. batava obscuriosa* adults, with an LC_50_ of 39.065 mg/L. The toxicity of other insecticides to adults of *R. batava obscuriosa* was between the above insecticides. The LC_50_ of 0.5% ivermectin, the LC_50_ of 10% lambda-cyhalothrin, and the LC_50_ of 6% spinetoram were 33.050, 37.016, and 38.309 mg/L, respectively.

As shown in Table 2, 48 h after application, the toxicity of the seven insecticides from strong to weak was 6% spinetoram, 10% broflanilide, 5% emamectin benzoate, 0.5% ivermectin, 10% abamectin·β-cypermethrin, 10% lambda-cyhalothrin, and 20% chlorfluazuron-thiamethoxam, with LC_50_ values of 4.973 mg/L, 5.459 mg/L, 5.486 mg/L, 9.481 mg/L, 10.641 mg/L, 15.210 mg/L and 18.042 mg/L respectively.

At 72 h, 10% broflanilide was the most toxic to *R. batava obscuriosa* adults (LC_50_ = 1.796 mg/L), followed by 6% spinetoram (LC_50_ = 2.294 mg/L) and 10% abamectin·β-cypermethrin (LC_50_ = 2.665 mg/L). Among the seven tested insecticides, 10% lambda-cyhalothrin exhibited the weakest toxicity to adult *R. batava obscuriosa* 72 h after application, with an LC_50_ of 14.059 mg/L. The toxicity of the other insecticides to adult *R. batava obscuriosa* was between those of the above insecticides.

#### 3.1.2. LT_50_ of Seven Insecticides Against Adults of *R. batava obscuriosa*

Based on the number of deaths of *R. batava obscuriosa* adults at different concentrations and time periods, the median lethal time regression equation and LT_50_ of the seven insecticides were obtained. As shown in Table 3, under the 500-fold dilution, the median lethal time of 10% abamectin·β-cypermethrin to *R. batava obscuriosa* adults was the shortest, with an LT_50_ of 7.564 h, indicating that the number of dead *R. batava obscuriosa* adults reached half at 7.564 h after application, and the quick-acting property was the best; the LT_50_ of 10% broflanilide under the 500-fold dilution was 9.585 h, second only to 10% abamectin·β-cypermethrin; the LT_50_ of 10% abamectin·β-cypermethrin under the other two concentrations was the smallest among the seven insecticides, and the lethal time was less than that of other insecticides.

### 3.2. Field Control Test of Four Insecticides Against R. batava obscuriosa

**Figure 3:**
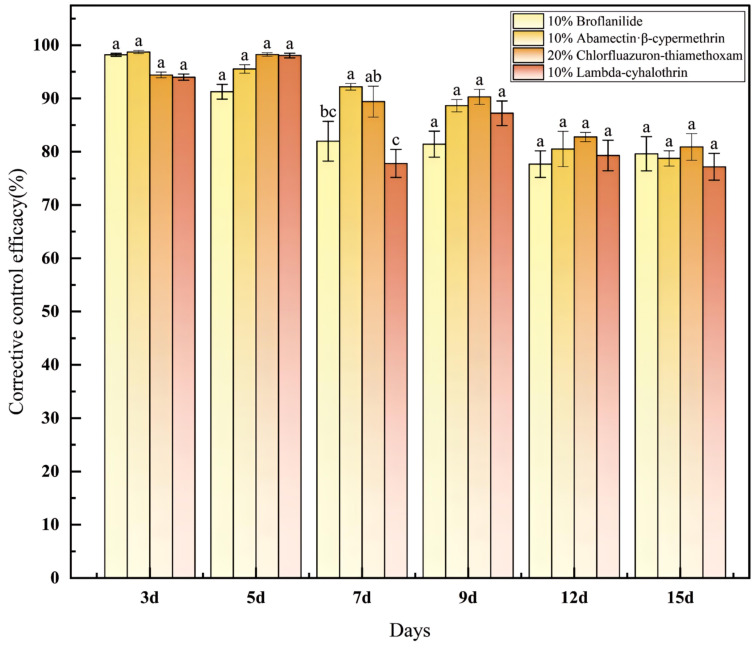
Evaluation of the control efficacy of four insecticides against *Rhagoletis batava obscuriosa*. Note: Different lowercase letters above the bars indicate significant differences in corrected control efficacy among different insecticide treatments at the same time point (*p* < 0.05, one-way ANOVA followed by Duncan’s new multiple range test). Data are presented as mean ± standard error (SE). The same letters denote no significant difference.

The field control effects of the four insecticides on *R. batava obscuriosa* are shown in Table 4 and Figure 3. Three days after application, all insecticide treatments resulted in mean trap catches that were significantly lower than that of the control (34 ± 2.52 individuals, *p* = 0.000), with no significant differences observed among the insecticide treatments themselves (*p* = 0.474). Three days after application, the corrected control effect of the four insecticides reached 94–98.73%. Five days after application, the corrected control effect exceeded 90%. The corrected control effect of 20% chlorfluazuron-thiamethoxam (98.25% ± 0.32%) and 10% lambda-cyhalothrin (98.06% ± 0.44%) demonstrated superior performance compared to 10% abamectin·β-cypermethrin.

From the efficacy of the four insecticides 7 days after application, it can be observed that 10% abamectin-chlorfenapyr exhibited the highest efficacy (92.19%), which was significantly higher than that of 10% broflanilide (*p* = 0.016) and 10% lambda-cyhalothrin (*p* = 0.002), but did not differ significantly from 20% chlorfluazuron·thiamethoxam (*p* = 0.454). The efficacy of 20% chlorfluazuron·thiamethoxam was significantly higher than that of 10% lambda-cyhalothrin (*p* = 0.008), while no significant difference was detected between 20% chlorfluazuron·thiamethoxam and 10% broflanilide (*p* = 0.062). Furthermore, 10% broflanilide and 10% lambda-cyhalothrin showed comparable efficacy (*p* = 0.268).

The corrected efficacy of 20% chlorfluazuron-thiamethoxam was the highest at 9 and 12 days after application, and the four insecticides were in the order of 20% chlorfluazuron-thiamethoxam > 10% broflanilide > 10% abamectin·β-cypermethrin > 10% lambda-cyhalothrin at 15 days after application.

**Figure 4:**
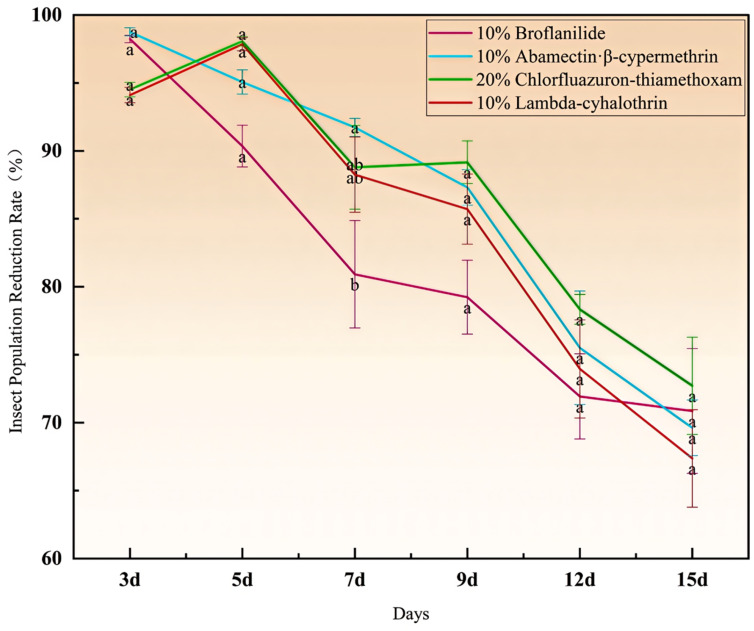
Insect population reduction rate of four insecticides after application. Note: Different lowercase letters above the data points indicate significant differences in insect population reduction rate among different insecticide treatments at the same time point (*p* < 0.05, one-way ANOVA followed by Duncan’s new multiple range test). Data are presented as mean ± standard error (SE). The same letters denote no significant difference.

The insect population reduction rate (Figure 4) of the four insecticides reached 94.1% to 98.76% at three days after application, with 10% abamectin·β-cypermethrin demonstrating superior efficacy (insect population reduction rate of 98.76%), followed by 10% broflanilide (insect population reduction rate of 98.23%). At 5 days after application, all insecticides achieved insect population reduction rates exceeding 90%. However, 20% chlorfluazuron-thiamethoxam (insect population reduction rate of 98.06% ± 0.36%) and 10% lambda-cyhalothrin (insect population reduction rate of 97.85% ± 0.49%) outperformed 10% abamectin·β-cypermethrin. On the 7th day after application, 10% abamectin·β-cypermethrin showed the best efficacy, with an insect population reduction rate of 91.73%, while the other three insecticides achieved insect population reduction rates exceeding 80% at 7 days, indicating overall good control efficacy.

Nine days after application, 20% chlorfluazuron-thiamethoxam had the highest population reduction rate (89.16% ± 1.57%), followed by 10% abamectin·β-cypermethrin, and 10% broflanilide had the lowest. At 12 days, 20% chlorfluazuron-thiamethoxam had the highest population reduction rate, followed by 10% abamectin·β-cypermethrin and 10% lambda-cyhalothrin.

In summary, all four insecticides effectively controlled the population of *R. batava obscuriosa*. Among them, 10% abamectin·β-cypermethrin has outstanding quick-acting properties, showing good effects within 3~7 days after application, but its long-acting properties slightly decrease over time; 20% chlorfluazuron-thiamethoxam has the strongest long-acting properties, with the highest control effect at 9~15 days after application and stable overall performance; 10% broflanilide has good both quick-acting and long-acting properties, and its control effect in the later period is superior to that of 10% lambda-cyhalothrin; 10% lambda-cyhalothrin has good quick-acting properties but slightly lower control effect in the later period compared to the other three insecticides. In controlling *R. batava obscuriosa*, these tested insecticides may be selected and used in rotation or combination based on the specific management needs of the pest.

**Figure 5:**
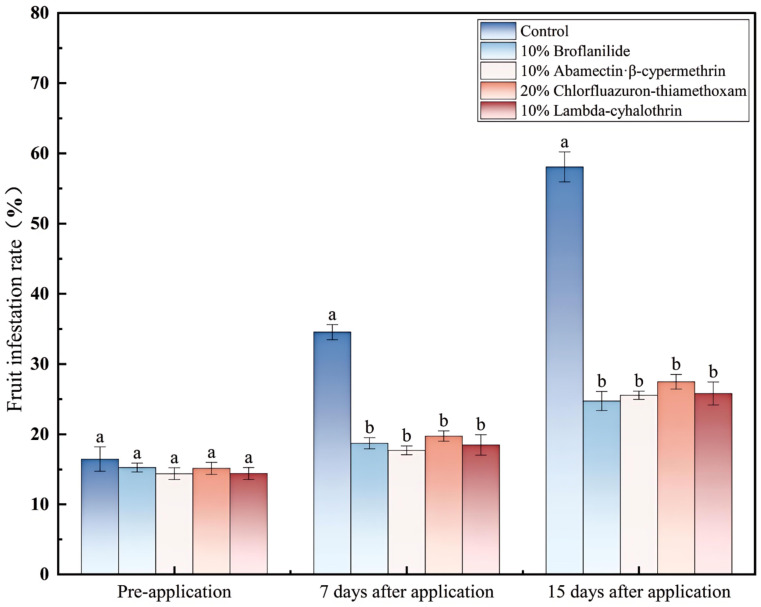
Reduction in fruit infestation rate of sea buckthorn by *Rhagoletis batava obscuriosa* after application of four insecticides. Note: Different lowercase letters above the bars indicate significant differences in fruit infestation rate among different insecticide treatments at the same time point (*p* < 0.05, one-way ANOVA followed by Duncan’s new multiple range test). Data are presented as mean ± standard error (SE). The same letters denote no significant difference.

As shown in Figure 5, 7 days after application, the fruit infestation rate of sea buckthorn in the control group was 34.56% ± 1.08%, which was significantly higher than that of the four insecticide-treated groups (*p* < 0.05). Among the treated groups, the 10% abamectin·β-cypermethrin group had the lowest fruit infestation rate (17.72% ± 0.62%), and the 20% chlorfluazuron-thiamethoxam group had the highest (27.49% ± 1.05%); however, there were no significant differences among the insecticide-treated groups. At 15 days after application, the fruit infestation rate of sea buckthorn in the control group was 58.08% ± 2.14%, which was significantly higher than that of the four insecticide-treated groups (*p* < 0.05). The 10% broflanilide group had the lowest fruit infestation rate (24.75% ± 1.36%), and the 20% chlorfluazuron-thiamethoxam group had the highest (27.49% ± 1.05%); however, there were no significant differences among the insecticide-treated groups (*p* = 0.338). The fruit infestation rate data demonstrated that all four tested insecticides significantly reduced damage to sea buckthorn fruits inflicted by *R. batava obscuriosa*.

## 4. Discussion

As an important economic tree species in northern China, the sea buckthorn industry is seriously threatened by *R. batava obscuriosa*. Chemical control remains the primary measure currently employed. Given the current lack of high-efficacy, low-toxicity insecticides and precise application technologies for controlling this pest, this study integrated laboratory toxicity determination with field efficacy evaluation and systematically assessed the toxicity of seven insecticides to adult *R. batava obscuriosa* via the residual film method. The results demonstrated that the seven tested insecticides differed significantly in toxicity at different time points, a finding closely associated with their modes of action and the physiological status of the test adults.

The toxicity determination results showed that 10% abamectin·β-cypermethrin had the strongest toxicity at 24 h, with an LC_50_ of 22.108 mg/L. This is basically consistent with the results of a study on the contact toxicity of four insecticides to adult *R. batava obscuriosa* [25], which showed that abamectin and the pyrethroid insecticide lambda-cyhalothrin had significant lethal effects on the pest. As a compound preparation of abamectin and beta-cypermethrin, the quick-acting advantage of 10% abamectin·β-cypermethrin may be due to the synergistic effect of the two components. Abamectin is a neurotoxin with stomach and contact toxicity to mites and insects [26], which mainly inhibits the conduction of nerve impulses in pests, causing paralysis and even death [27,28]. Beta-cypermethrin is a synthetic insecticide based on natural pyrethrins with stomach and contact toxicity [29], which mainly interacts with Na^+^ channels on the nerve membrane, affecting normal Na^+^ flow, thereby inducing toxic reactions [30,31]. The two components target different sites of the insect nervous system, thereby rapidly blocking nerve conduction and causing the test insects to die within a short time, further confirming the superiority of quick-acting compound insecticides.

A study on the toxicity of emamectin benzoate indicated that this insecticide is highly toxic to *Bactrocera dorsalis* adults [32]. The results of this study showed that the LC_50_ of 5% emamectin benzoate at 48 h was 5.486 mg/L. This may be because the effect of emamectin benzoate on interfering with glutamate-gated chloride channels is time-dependent. Within 48 h, the influx of chloride ions continued to increase, and neurological disorders (such as antifeedant and paralysis) gradually worsened, thereby improving the toxicity performance [33]. As a new type of antibiotic insecticide, emamectin benzoate mainly kills pests through stomach toxicity and has contact toxicity. It is characterized by a broad spectrum of activity, high-efficacy, low toxicity, low environmental residue, and low cross-resistance to conventional insecticides, making it widely utilized in pest management programs for vegetables, cash crops, and field crops [34,35]. Studies have shown that 72 h mortality and oviposition inhibition rates of *Anastrepha suspensa* under emamectin benzoate treatment reached 100%. Studies on stomach and contact toxicity of *B. dorsalis* have also shown that emamectin benzoate has a significant toxic effect on *B. dorsalis* [36].

In this study, the LC_50_ of 10% broflanilide at 72 h was significantly lower than that of the other insecticides, with an LC_50_ of 1.796 mg/L. This may be because broflanilide binds to ryanodine receptors in the endoplasmic reticulum of insect muscle cells, leading to the continuous release of calcium ions in the cells, ultimately causing muscle paralysis and death of the test insects [37]. This mode of action determines the persistence of its toxicity, which is consistent with the reported characteristic of long persistence of broflanilide in the control of *Tuta absoluta* [38].

Moreover, sea buckthorn groves are usually located in complex terrains with significant differences in their distribution among different regions. Traditional manual or ground mechanical application faces many challenges: traditional manual and mechanical application methods not only have high operational difficulty but also increase the risk of operators being exposed to harmful chemicals. Furthermore, ground machinery is limited by terrain and exhibits poor operational flexibility, making it difficult to ensure the uniform distribution of insecticides in the crown layer, thereby affecting the control effect.

Studies have shown that plant-protection UAVs can adapt to various crops and terrains, have high operational efficiency, and produce relatively low insecticide pollution. They are widely used to control pests and diseases in field crops, forests, and fruit trees [39,40,41]. Moreover, the coverage rate of UAV-based spraying in economic forests with complex terrain is significantly higher than that of manual spraying. In this study, the DJI Agras T20 plant protection UAV was used for field application, with an effective spray width of 7 m and an operating height controlled at 1.5 m–2 m above the sea buckthorn canopy. By precisely controlling the flight speed, height, and spray volume, uniform coverage of insecticide droplets on all parts of the sea buckthorn canopy was ensured, significantly improving the contact probability between the insecticides and adult *R. batava obscuriosa*.

A comparative study on insecticide application methods showed that compared with manual application, UAV application reduced the insecticide dosage by 30%, while the control effect was close to that of manual application [42]. Even reducing the dosage of insecticides for aerial control exerted a synergistic effect, greatly lowering the control cost. A study on UAV-based insecticide application for corn borer control demonstrated that combining UAV application with appropriate insecticides can significantly enhance the deposition efficiency of droplets on corn leaves [43]. Even under the condition of 20% water saving, the control effect reached an excellent level of 83%. A study on UAV-based control of *Spodoptera frugiperda* outbreaks in China found that the control effect gradually improved with the increase in water consumption, and the control effect under different spray volumes ranged from 59% to 85% [44].

Qinghe has a continental north temperate arid climate with dry air and a maximum summer temperature of 36.5 °C. The various insecticides screened through toxicity determination in this study had good control effects when applied using plant-protection UAVs in the field. However, it should be noted that environmental and weather factors, application dosage, application method, and the occurrence of *R. batava obscuriosa* can all affect the field control efficacy of the insecticides.

## 5. Conclusions

In Qinghe, *R. batava obscuriosa* causes severe damage, and the application of insecticides via UAVs has become the primary control measure. In this study, four insecticides targeting *R. batava obscuriosa* were screened through laboratory toxicity determination, and their field efficacies were evaluated. The results showed that the test insecticides had time-dependent effects on adult *R. batava obscuriosa*: 10% abamectin·β-cypermethrin showed good quick- and long-acting properties, the toxicity of 5% emamectin benzoate and 6% spinetoram significantly increased with time, and 10% broflanilide showed good long-acting properties.

The field trial results further validated the conclusions from laboratory toxicity tests: after spraying the four selected insecticides with a UAV, 7 days after application, 10% abamectin·β-cypermethrin showed the highest population reduction rate (91.73%) and corrected control effect (92.19%), reflecting its advantage in quickly controlling the pest population. The population reduction rates of the four insecticides at 7 days after application all exceeded 80%; at 15 days after application, the corrected control effects of the four insecticides reached 77.18–80.91%, and the fruit infestation rates were controlled within the range of 24.75–27.49%, confirming the precise and efficient characteristics of their chemical control. This indicates that these four insecticides have good continuous control effects on *R. batava obscuriosa* in the field. Simultaneously, it is recommended to reasonably select control insecticides, pay attention to the rotational use of insecticides, delay the occurrence of field resistance of *R. batava obscuriosa*, and improve the control effect.

## Figures and Tables

**Table 1:** Dilution Multiples of Seven Insecticides for Laboratory Toxicity Determination.

Insecticides	Dosage Form	Concentration Gradient (mg/L)				
10% Broflanilide	SC	3.75	7.5	15	30	60
10% Abamectin·β-cypermethrin	EW	15	30	60	120	240
20% Chlorfluazuron-thiamethoxam	SC	20	40	80	160	320
10% Lambda-cyhalothrin	EW	20	40	80	160	320
6% Spinetoram	SC	7.5	15	30	60	120
5% Emamectin benzoate	ME	7.5	15	30	60	120
0.5% Ivermectin	EC	7.5	15	30	60	120

**Table 2:** Laboratory toxicity of seven insecticides against *Rhagoletis batava obscuriosa* determined by the residual film method.

Time	Insecticides	Toxicity Regression Equation	Correlation Coefficient	Chi-Square Value	*p* Value	LC_50_ (mg/L)	95% Confidence Interval
24 h	10% Broflanilide	y = −1.43 + 1.01x	0.728	5.489	0.963	25.712	15.952~56.865
	10% Abamectin·β-cypermethrin	y = −1.06 + 0.79x	0.913	0.986	0.999	22.108	3.394~41.784
	20% Chlorfluazuron-thiamethoxam	y = −1.6 + 1.15x	0.928	3.055	0.998	26.237	11.875~40.028
	10% Lambda-cyhalothrin	y = −1.89 + 1.21x	0.819	4.136	0.990	37.016	17.985~56.475
	6% Spinetoram	y = −1.36 + 0.87x	0.708	4.912	0.977	38.309	20.847~88.919
	5% Emamectin benzoate	y = −1.81 + 1.14x	0.894	2.386	0.999	39.065	25.230~67.822
	0.5% Ivermectin	y = −1.34 + 0.88x	0.834	2.481	0.999	33.050	17.636~67.091
48 h	10% Broflanilide	y = −0.61 + 0.82x	0.895	1.257	0.999	5.459	0.993~10.100
	10% Abamectin·β-cypermethrin	y = −0.93 + 0.9x	0.912	1.16	0.999	10.641	0.829~22.568
	20% Chlorfluazuron-thiamethoxam	y = −1.4 + 1.2x	0.970	2.864	0.998	18.042	7.223~28.040
	10% Lambda-cyhalothrin	y = −1.32 + 1.23x	0.867	3.184	0.997	15.210	5.137~24.648
	6% Spinetoram	y = −0.5 + 0.85x	0.762	4.541	0.984	4.973	0.664~9.861
	5% Emamectin benzoate	y = −0.66 + 1.09x	0.860	3.604	0.995	5.486	1.621~9.215
	0.5% Ivermectin	y = −1.23 + 1.29x	0.829	4.768	0.980	9.481	4.386~14.387
72 h	10% Broflanilide	y = 0.03 + 1x	0.787	3.271	0.997	1.796	0.267~3.420
	10% Abamectin·β-cypermethrin	y = 0.11 + 0.58x	0.800	2.450	0.999	2.665	0.001~9.571
	20% Chlorfluazuron-thiamethoxam	y = −0.13 + 0.73x	0.675	3.127	0.997	6.868	0.298~15.280
	10% Lambda-cyhalothrin	y = −1.48 + 1.54x	0.714	2.667	0.999	14.059	4.793~20.239
	6% Spinetoram	y = 0.22 + 0.77x	0.794	4.522	0.984	2.294	0.041~5.251
	5% Emamectin benzoate	y = −0.31 + 1.09x	0.726	3.388	0.996	3.923	0.721~6.834
	0.5% Ivermectin	y = −0.55 + 1.26x	0.857	2.098	0.999	4.239	0.953~7.233

Note: For all Chi-square values in this table, *p* > 0.05 (goodness-of-fit test), indicating that the probit regression model fits the data well.

**Table 3:** LT_50_ determination of seven insecticides against *Rhagoletis batava obscuriosa*.

Insecticides	Dilution Multiple	Toxicity Regression Equation	Correlation Coefficient	Chi-Square Value	*p* Value	LT_50_ (h)	95% Confidence Interval
10% Broflanilide	500	y = −1.36 + 1.56x	0.912	3.486	0.996	9.585	4.303~13.953
	1000	y = −1.78 + 1.61x	0.953	5.027	0.975	14.102	8.543~18.914
	1500	y = −4.16 + 2.81x	0.928	6.344	0.933	29.817	24.284~35.716
10% Abamectin·β-cypermethrin	500	y = −0.81 + 1.23x	0.819	3.914	0.992	7.564	2.256~12.181
	1000	y = −1.57 + 1.49x	0.938	3.548	0.995	12.480	5.924~18.117
	1500	y = −2.6 + 2.01x	0.926	4.784	0.980	19.741	13.789~25.274
20% Chlorfluazuron-thiamethoxam	500	y = −1.37 + 1.40x	0.890	5.398	0.965	11.511	5.697~16.458
	1000	y = −2.42 + 1.90x	0.918	4.948	0.976	18.852	12.722~24.460
	1500	y = −2.02 + 1.72x	0.854	5.779	0.954	15.637	9.245~21.230
10% Lambda-cyhalothrin	500	y = −1.6 + 1.71x	0.958	2.899	0.998	10.369	5.208~14.613
	1000	y = −2.81 + 2.24x	0.947	3.75	0.994	18.018	12.946~22.702
	1500	y = −2.81 + 2.07x	0.883	6.917	0.906	22.505	16.474~28.346
6% Spinetoram	500	y = −3.25 + 2.69x	0.845	5.831	0.952	16.033	12.385~19.402
	1000	y = −3.07 + 2.46x	0.877	5.808	0.953	17.810	13.435~21.918
	1500	y = −2.9 + 2.04x	0.920	6.244	0.937	25.566	19.349~31.830
5% Emamectin benzoate	500	y = −4.25 + 3.54x	0.936	2.019	0.999	15.733	12.659~18.639
	1000	y = −4.66 + 3.44x	0.946	4.664	0.982	21.376	17.694~25.201
	1500	y = −4.25 + 2.85x	0.953	5.000	0.975	30.077	24.528~35.954
0.5% Ivermectin	500	y = −2.05 + 1.69x	0.829	7.959	0.846	16.756	10.750~22.106
	1000	y = −2.41 + 1.71x	0.810	9.812	0.709	24.575	17.734~31.329
	1500	y = −3.64 + 2.23x	0.876	11.95	0.532	38.057	31.153~46.021

Note: For all Chi-square values in this table, *p* > 0.05 (goodness-of-fit test), indicating that the probit regression model fits the data well.

**Table 4:** Field efficacy of four insecticides against *Rhagoletis batava obscuriosa*.

Group	Premedication Mean Catch	3 days After Application		5 days After Application		7 days After Application	
		Average Catch (Heads)	Corrective Control Efficacy (%)	Average Catch (Heads)	Corrective Control Efficacy (%)	Average Catch (Heads)	Corrective Control Efficacy (%)
Control	34.75 ± 1.44	34 ± 2.52 ^a^	——	38.25 ± 1.25 ^a^	——	36.75 ± 1.25 ^c^	——
10% Broflanilide	33.08 ± 1.02	0.58 ± 0.08 ^b^	98.2 ± 0.27 ^a^	3.17 ± 0.42 ^b^	91.27 ± 1.39 ^a^	6.25 ± 1.13 ^b^	81.98 ± 3.73 ^bc^
10% Abamectin·β-cypermethrin	34.58 ± 2.21	0.42 ± 0.08 ^b^	98.73 ± 0.30 ^a^	1.67 ± 0.22 ^b^	95.54 ± 0.8 ^a^	2.83 ± 0.08 ^a^	92.19 ± 0.62 ^a^
20% Chlorfluazuron-thiamethoxam	35.33 ± 2.68	0.67 ± 0.08 ^b^	94.41 ± 0.54 ^a^	1.92 ± 0.08 ^b^	98.25 ± 0.32 ^a^	3.83 ± 0.79 ^ab^	89.41 ± 2.91 ^ab^
10% Lambda-cyhalothrin	35.75 ± 2.32	0.75 ± 0.14 ^b^	94 ± 0.58 ^a^	2.08 ± 0.08 ^b^	98.06 ± 0.44 ^a^	4.08 ± 0.74 ^ab^	88.9 ± 2.62 ^c^
**Group**		**9 days After Application**		**12 days After Application**		**15 days After Application**	
		**Average Catch (heads)**	**Corrective Control Efficacy (%)**	**Average Catch (heads)**	**Corrective Control Efficacy (%)**	**Average Catch (heads)**	**Corrective Control Efficacy (%)**
Control		39.25 ± 3.07 ^a^	——	43.5 ± 4.63 ^a^	——	49.25 ± 5.98 ^a^	——
10% Broflanilide		6.83 ± 0.74 ^b^	81.42 ± 2.44 ^a^	9.25 ± 0.87 ^b^	77.68 ± 2.49 ^a^	9.58 ± 1.31 ^b^	79.62 ± 3.21 ^a^
10% Abamectin·β-cypermethrin		4.33 ± 0.22 ^b^	88.65 ± 1.17 ^a^	8.33 ± 1.08 ^b^	80.53 ± 3.32 ^a^	10.42 ± 0.08 ^b^	78.75 ± 1.44 ^a^
20% Chlorfluazuron-thiamethoxam		3.75 ± 0.25 ^b^	90.3 ± 1.41 ^a^	7.67 ± 0.79 ^b^	82.78 ± 0.87 ^a^	9.67 ± 1.62 ^b^	80.91 ± 2.51 ^a^
10% Lambda-cyhalothrin		5 ± 0.63 ^b^	87.23 ± 2.31 ^a^	9.17 ± 0.79 ^b^	79.3 ± 2.87 ^a^	11.5 ± 0.58 ^b^	77.18 ± 2.51 ^a^

Note: Data were analyzed using one-way ANOVA followed by Duncan’s new multiple range test. Different letters in the same column indicate significant differences at *p* < 0.05.

## Data Availability

The original contributions presented in this study are included in the article. Further inquiries can be directed to the corresponding author(s).
